# Surgical radioguidance with beta-emitting radionuclides; challenges and possibilities: A position paper by the EANM

**DOI:** 10.1007/s00259-023-06560-2

**Published:** 2024-01-08

**Authors:** Pedro Fragoso Costa, Kuangyu Shi, Soren Holm, Sergi Vidal-Sicart, Tereza Kracmerova, Giovanni Tosi, Jan Grimm, Dimitris Visvikis, Wolfram H. Knapp, Gopinath Gnanasegaran, Fijs W. B. van Leeuwen

**Affiliations:** 1 Department of Nuclear Medicine, University Hospital Essen, West German Cancer Center (WTZ), University of Duisburg-Essen, Essen, Germany; 2 Department of Nuclear Medicine, Inselspital, Bern University Hospital, University of Bern, Bern, Switzerland; 3 Computer Aided Medical Procedures and Augmented Reality, Institute of Informatics I16, Technical University of Munich, Munich, Germany; 4 Department of Clinical Physiology, Nuclear Medicine and PET, Rigshospitalet, University Hospital Copenhagen, Copenhagen, Denmark; 5 Nuclear Medicine Department, Hospital Clinic Barcelona, Barcelona, Spain; 6 Department of Medical Physics, Motol University Hospital, Prague, Czech Republic; 7 Department of Medical Physics, Ospedale U. Parini, Aosta, Italy; 8 Molecular Pharmacology Program, Memorial Sloan Kettering Cancer Center, New York, NY USA; 9 Department of Radiology, Memorial Sloan Kettering Cancer Center, New York, NY USA; 10 UMR 1101, LaTIM, INSERM, University of Brest, Brest, France; 11 Department of Nuclear Medicine, Medizinische Hochschule Hannover, Hannover, Germany; 12 Institute of Nuclear Medicine, University College London Hospital, Tower 5, 235 Euston Road, London, NW1 2BU UK; 13 Royal Free London NHS Foundation Trust Hospital, London, UK; 14 Interventional Molecular Imaging Laboratory, Department of Radiology, Leiden University Medical Center, Leiden, the Netherlands

**Keywords:** Beta emitting Radionuclides, Radioguidance, Radiotracers, Image guided surgery, Radiation exposure

## Abstract

Radioguidance that makes use of β-emitting radionuclides is gaining in popularity and could have potential to strengthen the range of existing radioguidance techniques. While there is a strong tendency to develop new PET radiotracers, due to favorable imaging characteristics and the success of theranostics research, there are practical challenges that need to be overcome when considering use of β-emitters for surgical radioguidance. In this position paper, the EANM identifies the possibilities and challenges that relate to the successful implementation of β-emitters in surgical guidance, covering aspects related to instrumentation, radiation protection, and modes of implementation.

## Introduction

Despite the rapid advance of alternatives, surgery remains to be one of the key treatment pillars for oncological care. Not only does it enable radical removal of diseased tissue, it is also one of the cheapest treatment options available. Surgery is also increasingly being used in a neoadjuvant setting where it provides complementarity to chemo-, immune-, or (external beam) radiation therapy. The nuclear medicine disciplines of radioguided surgery and interventional nuclear medicine provide an ever-increasing armory of technologies that support precision interventions. At the same time, within nuclear medicine, the diagnostic use of PET is rapidly advancing the field of molecular imaging [1, 2]. A success has resulted in the widespread clinical availability of β^+^-emitting PET tracers. Many recent theranostic research activities focus on providing therapeutic β^−^-emitting analogues of these radiotracers, an example being the theranostic pair of [^68^ Ga]Ga-PSMA-11 and [^177^Lu]Lu-PSMA-617 [3]. The success of these efforts has also stimulated an interest in the use of β-emitting tracers for image guided surgery purposes. β-surgical radioguidance can essentially take place via four routes: (1) direct detection of the β^+^ or β^−^ particles [4], (2) direct gamma emissions (prompt or concurrent), (3) detection of secondary 511 keV annihilation photon emissions [5], and (4) detection of secondary Cerenkov light emissions arising from the emitted positron (and electron) [6]. In theory, these methodologies can complement each other, but in practice, they are often used individually.

**Fig. 1:**
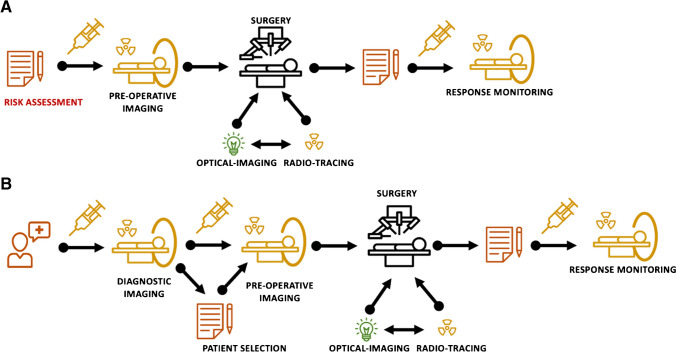
Surgical radioguidance workflows*.* General workflows in surgical radioguidance: **A** sentinel node procedures and **B** receptor targeted procedures

The current clinical standard in radioguidance (Fig. 1A) is set by the well-documented, well-accepted, and well-validated use of low to mid-energy γ-emitters, Gamma-radioguidance. Low dose exposure and ready availability of compatible detectors and cameras have driven the use of ^125^I/^123^I, ^111^In, and in particular ^99m^Tc for radioguided surgery [7–9]. A clear example is the routine and wide scale use of [^99m^Tc]Tc-radiocolloids during sentinel node (SN) procedures [10, 11], where preoperative imaging creates a (3D) roadmap that surgeons can use to navigate toward lesions in unusual and unexpected locations. Following this macroscopic navigation, the lesions are then detected with tailored surgical modalities [12]. Overall, however, radiochemical efforts in the area of SPECT tracer development seem to be lacking behind in popularity due to the superior imaging characteristics of PET imaging modalities. Making it logical to study to what extent these imaging guidance aspects could potentially be covered via β-surgical radioguidance.

While there are clear arguments to be made in favor of using β-emitting radionuclides, not one β-surgical radioguidance strategy has made it to routine clinical use to date. The popularity of the topic is thus mainly based on technology-driven clinical trials that provide a limited body of evidence. This leads to unclarity within the nuclear medicine and surgical communities. The aim of this position paper is to provide a critical overview on the available technologies and the chances and challenges that come with β-surgical radioguidance. This is done by discussing β-radioguided strategies relative to the $\gamma$-surgical radioguidance paradigms currently in clinical use (Fig. 1A vs B), thereby addressing aspects such as cost calculations, logistics, and surgical imaging equipment, radiation exposure for surgical staff and patients, and ethics. From a medical perspective, these factors raise some questions. For example, are the new technologies valid alternatives and if so, under which conditions should they be applied?

## Logistical aspects of radioguidance workflows

Nuclear medicine imaging technologies are increasingly being used to classify oncologic patients and to select patients for therapy, including surgery. Hereby, PET is generally seen as the preferred diagnostic modality; the sensitivity and spatial resolution of PET imaging are superior to those of SPECT [13]. Diagnostic PET scans help physicians in selecting patients with local disease that are most likely to benefit from surgical tumor removal. That said, there often is a period of weeks or months in between the initial PET scan and the surgery itself. The possibility of extended tumor growth in this period makes it imperative to generate a second nuclear imaging scan 1 or 2 days before surgical radioguidance. These secondary scans substantiate patient restaging and help create an up-to-date roadmap for surgical guidance (Fig. 1B). A clear example here is PSMA gamma-radioguided salvage nodal surgery that is in clinical trials, but also is now routinely implemented outside of clinical trials in some European hospitals [14]. While patient selection is based on a diagnostic PSMA-PET scan, the selected patients undergo a second [^99m^Tc]Tc-PSMA SPECT scan on the day of surgery [15]. β-surgical radioguidance trials that use Cerenkov-based PSMA imaging essentially follow the same paradigm, meaning that in this application, patients tend to get a second PSMA-PET scan at the day of surgery [16]. This requirement for an additional imaging session creates some logistical challenges, whereby the time between imaging and surgery, the physical half-life of the radionuclide, and the pharmacokinetics of the radiotracer are the most critical factors.

In SN mapping with ^99m^Tc (T_1/2_ = 6 h), procedures are performed in 1 day (injection and imaging in the morning and surgery in the afternoon) or 2 days (injection and imaging in the afternoon and surgery the following morning) protocols [17]. Initially, PET analogies of these procedures therefore made use of the much more costly ^89^Zr (T_1/2_ = 78.4 h) to maintain the clinical logistics [18]. Where SN procedures only require 1–2 h to visualize the targets following local injection, applications with intravenously injected receptor-targeted tracers tend to require much longer to clear background signals. A prominent example herein is the 12-h time interval needed between radiopharmaceutical administration and [^99m^Tc]Tc-PSMA-I&S-guided surgery [9, 19]. While relatively long-living radionuclides can easily accommodate these workflows, this poses challenges for radionuclides with short half-lives. For example, SN approaches (Fig. 1A) that use short-living PET radionuclides such as [^68^ Ga]Ga-tilmanocept (T_1/2_ = 1.1 h) [20] or 2-[^18^F]FDG (T_1/2_ = 1.8 h) [21] require the imaging to be performed shortly before surgery, which calls for complex coordination. The same applies for receptor targeted applications (Fig. 1B) such as [^68^ Ga]Ga-PSMA [16, 22] and could be extended to tracers such as [^18^F]F-SiFAlin-TATE [23].

The most straightforward way to ensure signals can still be detected during surgery would be to increase the activity injected, but this would increase the radiation exposure for the surgical staff. There is still lack of evidence to which minimal patient administered activity renders a measurable signal for typical surgery durations.

Approaches that rely on β-radioguidance with longer-lived radionuclides such as ^90^Y (T_1/2_ = 2.7 d) could provide reasonable signals with much lower administered activities, such is the case of a β-surgical radioguidance feasibility study using [^90^Y]Y-DOTA-TOC [4], but still require particular attention in terms of extremities exposure, due to the high energy of beta particles emitted in ^90^Y decay.

The settings of the surgery itself can vary substantially between indications. The most common approaches in surgical radioguidance include “open” surgery [24], laparoscopic surgery [25], and robot-assisted laparoscopic surgery [26]. The performance of the radiotracer is not affected by the surgical approach, but the approach does directly reflect the positioning of the surgical staff, the accessibility of the imaging modalities used, and the degrees of freedom with which these modalities can be used. In particular, “key-hole” procedures demand dedicated miniaturized (small and lightweight) and preferably steerable tools. This feature tends to directly impact detector design features such as ergonomics, collimation, and sensitivity. An example here is the development and evolution of laparoscopic gamma probes [27].

Given that surgeries generally comprise multiple facets, whereby only one is the radioguided resection, the duration of image acquisition is essential. In practice, there is a substantial demand for real-time feedback [27]. That said, static intraoperative overview imaging with, e.g., portable gamma cameras have also proven valuable [28]. Ex vivo or rather “back table” assessments using cameras and/or tracing probes add value by confirming that lesions have been accurately resected [29]. A unique characteristic for these back table assessments is that they do not physically restrict the use of a specific type of modality.

It is important to note that some surgeries take substantially longer than others, meaning the surgical staff spends more time next to “hot” patients or that the radioactive signal may decay. It is not uncommon for a procedure to take 5 h. In the case of a robot-assisted operation, the surgeon is out of harm’s way. Meaning he/she is not exposed to the radiation provided by a β^+^-emitting radiopharmaceutical. Still, the bedside assistant is continuously being exposed to the radiation from radioactivity residing in the patient or the resected specimens.

## Clinical targets and radiotracers

Today the most common clinical target benefitting from surgical radioguidance still is the SN [30–32], followed by radio-occult lesion localization (ROLL) [33], iodine seed marking [34], and thyroid surgery [35]. The treatment of neuroendocrine tumors has also been pursued using somatostatin receptor-targeted radioguidance strategies [36]. PSMA-targeted surgery, however, seems to be the procedure that is on the rise at the moment [37]. As depicted in Table 1, there is a PET alternative for most SPECT tracers and vice versa. Indeed, up-and-coming approaches such as FAPI-PET have SPECT analogues available [38]. The only tracer for which there is currently no optimal SPECT alternative is 2-[^18^F]FDG. While tracers such as [^99m^Tc]Tc-Sestamibi also depict some form of metabolism, their performance does not equal that of 2-[^18^F]FDG. Given the widespread implementation of 2-[^18^F]FDG in oncological imaging (including diagnostic, staging, restaging, and therapy monitoring), there is a clear window of opportunity for β-surgical radioguidance based on 2-[^18^F]FDG.

**Table 1:** Clinical targets and radiotracers used in patients. A comprehensive overview of the clinical application of nuclear detection methods in surgical radio guidance can be found in [27]

Application	β-particle emitting radiotracer	$\gamma$-photon emitting radiotracer
Sentinel node	[^68^ Ga]Ga-tilmanocept [^89^Zr]Zr-nanocolloid	[^99m^Tc]Tc-nanocolloid ICG-[^99m^Tc]Tc-nanocolloid [^99m^Tc]Tc-tilmanocept [^99m^Tc]Tc-sulfur colloid [^99m^Tc]Tc-phytate colloid [^99m^Tc]Tc-rhenium colloid [^99m^Tc]Tc-Senti-Scint (HSA colloid) [^99m^Tc]Tc-antimony-trisulfide
Thyroid	[^124^I]I-NaI [^123^I]I-NaI^a^ [^131^I]I-NaI	[^125^I]I-NaI [^123^I]I-NaI [^131^I]I-NaI
Iodine seeds	^n.a^	^125^I
PSMA overexpression	[^68^ Ga]Ga-PSMA-11 [^18^F]PSMA-1007	[^99m^Tc]Tc-PSMA I&S [^111^In]In-PSMA I&T [^111^In]In -PSMA-617
Tumor metabolism	2-[^18^F]FDG	[^99m^Tc]Tc-Sestamibi [^99m^Tc]Tc-Tetrofosmin
Somatostatin receptor overexpression	[^90^Y]Y-DOTA-TOC [^68^ Ga]Ga-DOTA-TOC [^68^ Ga]Ga-DOTA-NOC [^68^ Ga]Ga-DOTA-TATE	[^99m^Tc]Tc-Demotate [^99m^Tc]Tc-EDDA/HYNIC-TOC [^111^In]In -pentetreotide [^125^I]I-Tyr^3^-octreotide [^125^I]I -lanreotide

^a^Mono-energetic conversion electrons

From a radiopharmaceutical perspective, the radiochemical efforts that are specifically geared towards surgical radioguidance are focused on generating dual-labelled, bimodal, or rather hybrid radiotracers. A concept wherein radioguidance approaches are further strengthened through the introduction of intraoperative fluorescence guidance [39]. In this setting, both PET/fluorescence and SPECT/fluorescence approaches are being pursued in platforms ranging from small molecules to peptides, proteins, monoclonal antibodies, and even nanoparticles [40–42]. As with the conventional radioguidance approaches, the clinical implementation of these concepts is driven by a SPECT/fluorescent tracer for SN procedures (indocyanine green (ICG)-[^99m^Tc]Tc-nanocolloid) [43].

## Modalities and mode of implementation

Given the different settings and routes through which β-emissions can be imaged or traced during surgery, a wide range of beta-ray imaging/tracing modalities has been reported [27]. These modalities and their implementation are summarized in Table 2.

**Table 2:** Surgical modalities and their mode of clinical implementation. An extensive review of Clinical application of nuclear detection modalities in surgical radioguidance is available in [27]

	Open surgery	Endoscopic surgery	Laparoscopic surgery	Robot-assisted surgery	Back table examination
511-keV annihilation photons probe	2-[^18^F]FDG Colorectal cancer, breast cancer, lymphoma, ovarian cancer [^18^F]F-L-DOPA Brain tumors [^124^I]I-cG250 Renal [^68^ Ga]Ga-DOTA-NOC/TATE Neuroendocrine tumors	2-[^18^F]FDG Esophageal carcinoma	2-[^18^F]FDG Ovarian cancer Lung cancer	NA	NA
β probe	2-[^18^F]FDG Breast cancer, tongue tumor ^32^P buffered phosphate ion solution Glioma [^124^I]I-cG250 Renal cell carcinoma [^90^Y]Y-DOTA-TOC Meningioma Neuroendocrine tumors	NA	NA	[^68^ Ga]Ga-PSMA-11	2-[^18^F]FDG Renal cancer [^68^ Ga]Ga-PSMA positive Primary PCa and LN metastasis
Cerenkov imaging	NA	2-[^18^F]FDG positive colon lesions Hepatocellular carcinoma	NA	NA	[^68^ Ga]Ga-PSMA positive primary PCa margins 2-[^18^F]F-FDG breast cancer
PET/CT-based surgical navigation	NA	NA	2-[^18^F]FDG Intrathoracic lesions Bone lesions Lymph nodes	NA	NA

PCa: prostate cancer LN: lymph node

### Positron annihilation-photon (γ) probes

**Fig. 2:**
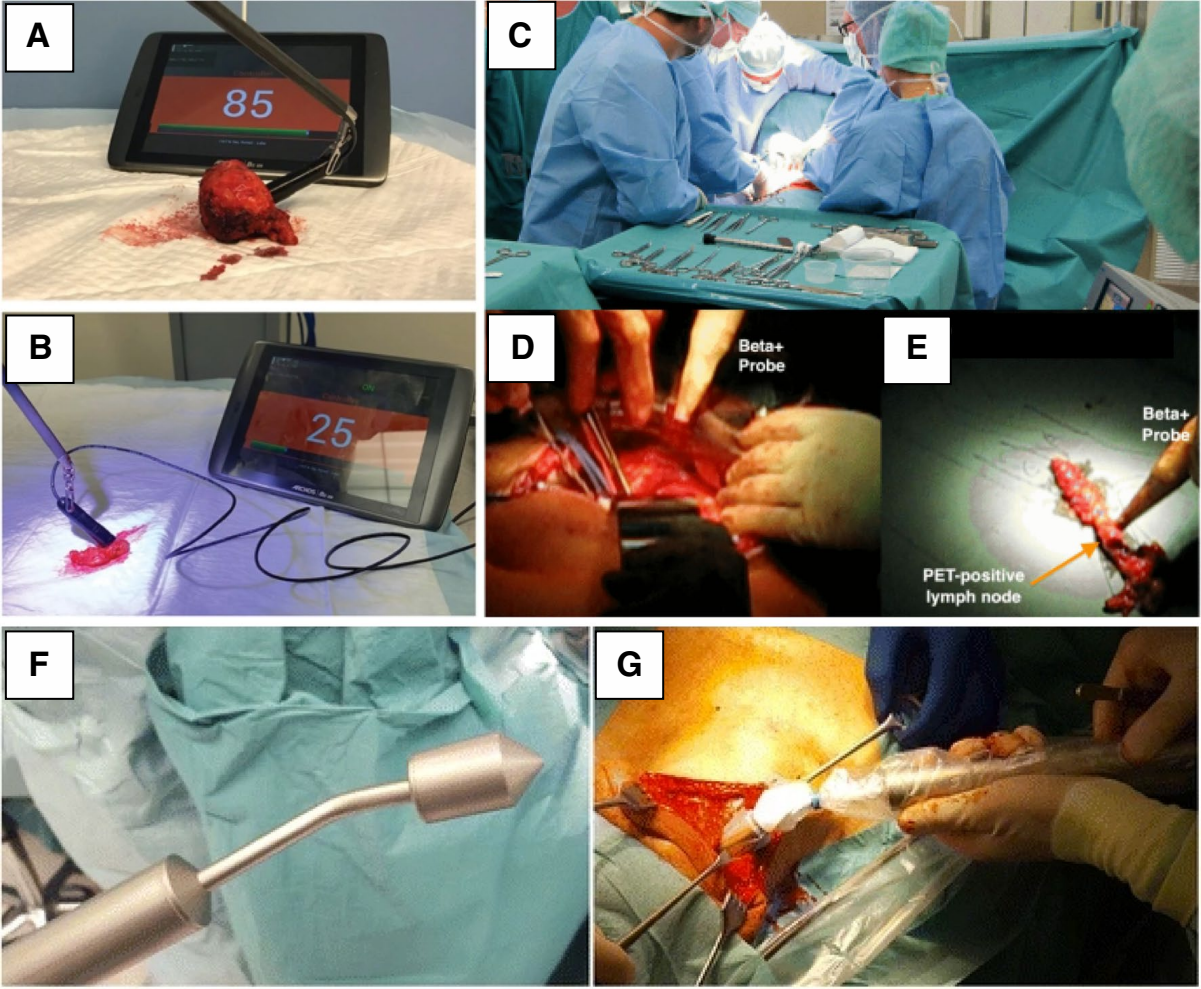
Intraoperative use of beta-tracing probes. Example of robot-assisted beta-tracing with the DROP-IN beta probe on the surface of a resected prostate sample (**A**) and resected lymph node package (**B**) in prostate cancer specimens after injection of [^68^Ga]Ga-PSMA-11 [45]. Surgical ablation and ex vivo verification of the metastatic lymph node with 2-[^18^F]FDG (**C**–**E**) [46]. Intraoperative detection of sentinel lymph nodes with [^89^Zr]Zr-nanocolloid-albumin PET-CT using a handheld high-energy gamma probe (**F**–**G**) [18]

High-energy $\gamma$ probes are scintillation detectors such as bismuth germanate (BGO) and lutetium orthosilicate (LSO) designed to detect positron annihilation 511 keV photons [44]. In this energy, the signal attenuation by tissue is limited, meaning deeper lesions can be identified. Unfortunately, this also means that distant signals may also cause high background. To provide enough stopping power for efficient signal collection, scintillation crystals need to be of a sufficient thickness (typically > 2 cm). Perhaps the greatest challenge for 511 keV annihilation-photon-probes is the need for substantial collimation (Fig. 2F). Such collimation is needed to allow focal target identification. Unfortunately geometric collimation of high-energy photons can compromise ergonomics, because of size (probe-head diameters of about 25 mm) [47] and weight (weights up to 500 g, Fig. 2F, G) [18]. Further developments in 511 keV surgical radioguidance will probably aim to optimize detector-collimator design [24]. For example, a multi-detector setup has been developed to perform electronical collimation of signals of 511 keV annihilation photons at a reduced weight, but still requiring a rather large diameter of ~ 30 mm [27].

### Beta plus (β^+^) and beta minus (β^−^) probes

For most β^+^ /β^−^ emitters, the radiopharmaceutical is administered systemically, which presents a challenge in attaining a conspicuous lesion signal from the high uptake on background tissues. This can be solved by only detecting β^+^ /β^−^ emitted particles that travel < 2 mm through tissue. Physics restraints limit the in depth detection and the effective measurement volume, meaning this superficial approach is highly reliant on preoperative road maps [48]. Ideally, scintillator-based beta probes should be insensitive to annihilation photons and at the same time provide a maximum light yield for charged particles. Plastic scintillators or organic scintillators (e.g., p-terphenyl) can be used in relatively small (10 mm in diameter and 150 mm in length) and lightweight (< 100 g) designs (Fig. 2D, E) [44, 46, 49]. Minimal geometric collimation using steel or plastic material is often sufficient due to the short-range attenuation of β-particles [4, 50]. For pure β^−^ measurements, photon background due to bremsstrahlung is usually negligible because of its very low emission probability [4]. However, pure β^+^ measurements are always interfered by annihilation high-energy photons [51]. A dual-detector design is typically employed to correct the mixed β^+^ and annihilation photons signal by additionally measuring the signal arising from annihilation photons using a second detector behind the main detector [50, 52]. Most β^+^/ β^−^ probes have been designed and used in open surgical applications [18, 24, 27, 46, 50, 52]. Further developments in probe design have allowed compatibility with laparoscopic surgery [53] or robotic surgery [45], including small and flexible fiberscopic β^+^ imaging probes [25]. Of note, lesion identification via rigid laparoscopic guidance modalities suffers from a loss of rotational freedom, while tethered/DROP-IN modalities can accommodate lesion identification across the full range of motion of the surgical platform [54].

### Beta cameras

Beta cameras provide a similar function to that of a beta probe, with the ability to provide a 2D representation of the specimen by means of pixelated detectors [55, 56]. Some implementations can be using dual-detector design [55, 57], charged-coupled detector (CCD) with scintillator [58], or complementary metal oxide semiconductor (CMOS) [59, 60]. The dual-detector beta cameras use two stacked scintillation materials in different depths to tackle the challenge of high-rate background. It can discriminate the superficial β-signal from the deeper penetrating annihilation photons. Subsequent photon signal subtraction allows for a representation of the signals originating from β-particles only [61]. Recent developments on semiconductor detector technology may facilitate the direct measurement of β^+^ and β^−^ signal without scintillation material [62, 63]. This yields a beta camera that has a high spatial and contrast resolution and is insensitive to background annihilation photons [64]. The recorded energy information can be further used to correct the background scattering and enhance the spatial resolution [65].

### Optical Cerenkov luminescence imaging

**Fig. 3:**
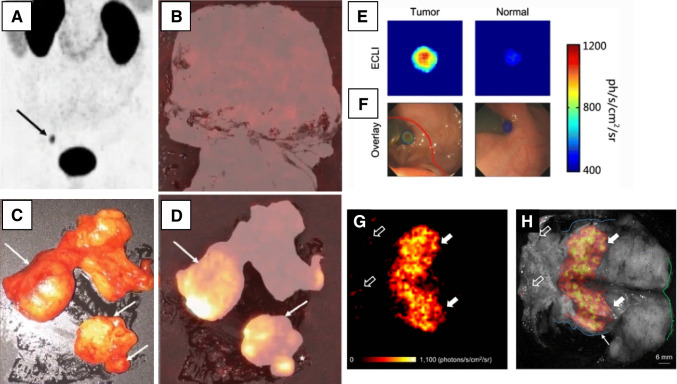
Intraoperative use of Cerenkov luminescence imaging. Cerenkov luminescence imaging for oligometastatic prostate cancer after [^68^ Ga]Ga-PSMA-11 PSMA-PET/CT (**A**) indicating prostate tumor close to the surface of the organ. Example of Cerenkov imaging detecting no tumor close to the surface of the resected prostate (**B**). Macroscopic lymph node metastasis and two different nodes were palpable (**C**, arrows). Corresponding Cerenkov imaging detecting PSMA-positive lymph nodes (**D**) [70]. Endoscopic Cerenkov luminescence imaging, after injection of 2-[^18^F]FDG, showing cancerous lesions of the GI tract (**E**, left panel) and overlaid with white-light images taken by clinical colonoscopy (**F**) [71]. No special type invasive breast cancer Cerenkov image using 2-[.^18^F]FDG (**G**) and gray-scale photographic image overlaid with Cerenkov signal (**H**) showing increased signal from tumor is visible (white arrows) [72]

Cerenkov luminescence (CL) is emitted when a charged subatomic particle, for example, from β decay, traverses a medium (typically up to 1–2 mm, depending on the positron energy [66]) with a velocity that exceeds the in-medium phase speed of light [6, 67]. The Cerenkov luminescence imaging (CLI) spectrum predominantly comprises a peak intensity in the ultraviolet light with a tail-emission extending up to far-red end of the light spectrum [68]. Recently, even a short-wave infrared (SWIR) component of CL was demonstrated [69]. Like β-particle detection, CLI is limited to detection of signals emitted in superficial tissue layers (Fig. 3) [6] and imaging of sources underneath 1 mm of scattering medium can be performed with a spatial resolution of 2.4 and 2.7 mm, for ^18^F and ^68^ Ga, respectively [73]. Hereby superficial light emissions will overshine more diffuse light signal coming from deeper lesions. Without overlying signals, it has been shown that it is possible to detect deeper lying lesions [69]. This also raises a key concern for CLI, namely detection of other light sources or even ambient light.

The CLI imaging systems are usually using cooled detectors (− 90 °C) such as light sensitive charged couple devices (CCDs) [6, 74]. The architecture for CLI detectors is dominated by maximizing the sensitivity. The efficiency of the Cerenkov light-conversion is about 3 orders of magnitude lower for ^18^F when compared with a typical surgical dye such as indocyanine green (ICG) [75]. To compensate for the low light intensity, cameras tend to collect signal over a wide spectral range and require longer acquisition time (typically about 5 min per view) to collect enough signals to create an image [76]. As Cerenkov signals are prone to contamination by other light sources, CLI imaging must be performed in a setting that blocks out any interfering light. For example, by creating an ambient light-tight environment [77].

### Three-dimensional detection modalities

As stated before, it is common that 3D SPECT or PET roadmaps are used during surgical procedure planning and are retrieved during surgery. Current extended reality display options now allow for pre-interventional scans to be integrated into the surgeons’ (endoscopic) view [78], thus supporting a navigation workflow. The biggest challenge for such navigation strategies are the tissue deformations that occur during the surgical intervention and the resulting mismatch with the original SPECT or PET data sets [79]. To overcome such challenges, technologies have been developed that reconstruct 3D imaging from intraoperative gamma or beta imaging probes or cameras, for example, the Freehand SPECT [80–82], freehand beta [80], and intraoperative PET imaging from high-energy $\gamma$ probes [83].

**Fig. 4:**
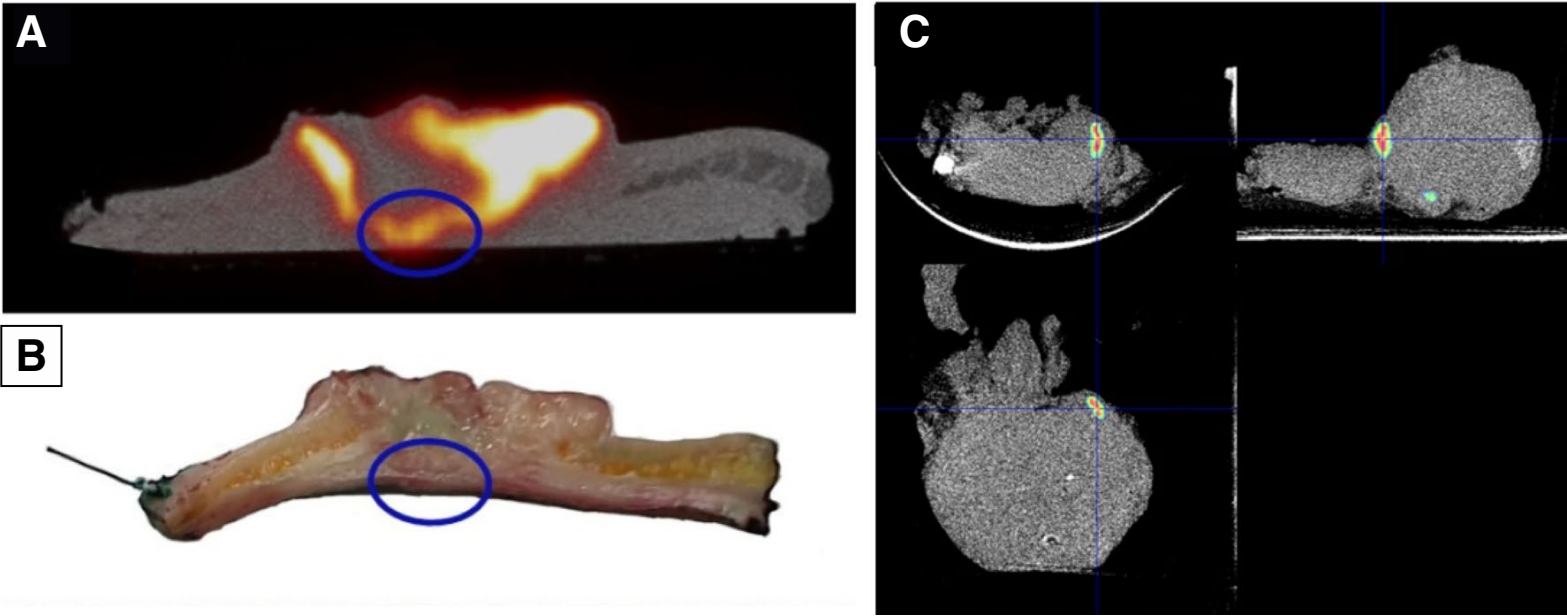
PET/CT imaging in the assessment of intraoperative margins*.* Back table PET/CT imaging, after 2-[^18^F]FDG administration, of a cutaneous squamous cell carcinoma (**A**) and the sliced tissue of the proposed region corresponding with the PET/CT imaging (**B**) [86]. PET/CT imaging of the prostate preparation after preoperative [^18^F]PSMA-1007 injection with evidence of tracer-uptake immediately at the resection margin (**C**) [84]

Another three-dimensional intraoperative approach consists of using small-bore PET/CT scanners in ex vivo or “back table” assessments of surgical resected specimens (Fig. 4). High-resolution preclinical scanners, with depth of interaction correction, can achieve a spatial resolution of about 1.0 mm using ^18^F. The model-based iterative reconstruction algorithms can estimate the 3D location of each interaction within the PET detectors thereby diminishing the parallax effect, which results in an optimization of the spatial resolution [85]. Furthermore, it allows for the integration of CT-based anatomical information. The main drawbacks of this modality are long acquisition times (30 min), extremely limited portability, and image to patient registration [86].

### Future engineering developments

On top of the above-mentioned imaging modalities for β-emitting radionuclides, one may envision that-similar to what we have seen for γ-guidance-hybrid modalities will be developed that integrate other intraoperative imaging methods such as ultrasound [87] or fluorescence imaging [88–90]. With the advancement of robotic surgery, the development of matching surgical radioguidance modalities provides interesting possibilities. It includes the development of purpose tailored probes or cameras such as the DROP-IN beta probe [45] (Fig. 2A, B) or EndoTOFPET [91]. Finally, the registration and visualization of intraoperative nuclear medicine imaging with robotic imaging can further enhance minimally invasive robotic surgery [92].

## Limited clinical evidence

The current body of evidence referring to clinical trials on β-surgical radioguidance is mostly based on alternative use of approved radiopharmaceutical (i.e., 2-[^18^F]FDG, [^68^ Ga]Ga-DOTA-TOC, and [^68^ Ga]Ga-PSMA). In general, these technologies aim at confirming the technical feasibility, mostly using histopathology staining as reference. Table 3 summarizes completed, active, and recruiting registered clinical trials (available from www.clinicaltrials.gov) that evaluate β-surgical radioguidance.

**Table 3:** Current registered clinical trials available from www.clinicaltrials.gov (accessed 20 October 2023)

Study start/completion date	Country	ID	Official title	Status	Primary objective	Enrolment	Reference
10.2008/08.2011	USA	NCT00816335	Pilot study evaluating a combined and integrated technological approach of F-18-FDG-Directed perioperative PET/CT imaging and intraoperative handheld gamma prove detection of known and occult disease in patients undergoing surgery for solid malignancies	Completed	Determine if perioperative PET/CT imaging combined with intraoperative use of handheld gamma probe is feasible during surgical procedure to resect malignancy	65	[93]
01.2012/03.2014	Canada	NCT01467219	Intra-operative lymph node evaluation using a hand-held PET gamma probe in endometrial cancer surgery—a pilot study	Completed	Identification of metastatic disease in endometrial cancer through pre-operative PET assessment in combination with an intra-operative gamma probe	12	[94]
08.08.2012/03.04.2018	USA	NCT01664936	Non-Invasive Cerenkov luminescence imaging of lymphoma, leukemia and metastatic lymph nodes	Completed	Assess the feasibility of clinical Cerenkov luminescence imaging using current clinical radiotracers (2-[^18^F]FDG and [^131^I]I-NaI) and a highly sensitive camera	27	[95]
03.2013/12.2016	USA	NCT01826227	Intraoperative detection of lesions using PET (Positron Emission Tomography) probe during secondary cytoreductive surgery for recurrent ovarian, fallopian tube and primary peritoneal cancer: a pilot study	Completed	Sensitivity of detection of lesions with PET probes compared to preoperative 2-[^18^F]FDG PET and standard intraoperative examination. Sensitivity is defined as the percent of lesions that were found with malignant disease divided by the number of lesions with true presence of malignant disease based on the pathology report	5	None
06.2014/06.2016	United Kingdom	NCT02037269	A pilot study to evaluate Cerenkov luminescence imaging for measuring margin and lymph node status in breast cancer surgical specimens	Completed	Tumor margin status of the wide local excision specimen determined by the imaging system	25	[72]
16.02.2017/31.12.2019	Italy	NCT04296149	Beta probe and surgery in GEP NET: evaluation of a new probe (RADIONET)	Completed	Evaluation of sensitivity of a beta probe	5	[96]
18.08.2017/13.11.2019	USA	NCT03213951	Ex vivo investigation of beta probe for prostate cancer resection and evaluation of PSMA-PET for diagnosis of intraprostatic lesions	Completed	Assess beta ray detection of prostate cancer ex vivo using experimental beta probe immediately after prostate cancer removal as determined by pathologist	28	[97]
20.03.2018 /	USA	NCT03484884	A feasibility study of non-invasive Cerenkov luminescence imaging in patients with cancer	Active, not recruiting	Explore the overall feasibility of clinical Cerenkov imaging on patients with any tumors with nodal metastases (existing or suspected) scheduled for routine clinical 2-[^18^F]FDG PET or [^131^I]I-NaI therapy	102	[77]
28.08.2020/30.09.2022	Belgium	NCT05068687	Perioperative assessment of tumor resection margins using high-resolution ^18^F-FDG-PET/CT in malignancies of the head and neck, a pilot study	Completed	Determine margin status in malignancies of the head and neck To investigate the ability of high-resolution 2-[^18^F]FDG PET/CT-scan to determine the margin status in malignancies of the head and neck. This will be compared to the gold standard of histopathological examination	9	[86]
21.03.2022 /	Italy	NCT05596851	PSMA radio-guided lymph node dissection with a beta probe, in high-risk prostate cancer patients	Recruiting	To evaluate the diagnostic accuracy of the combined approach with β probe and [^68^ Ga]Ga-PSMA-11 PET/CT in the correct identification of lymph node metastases, in high-risk prostate cancer patients undergoing radical prostatectomy and pelvic lymph node dissection. The histopathological analysis of the surgical specimens will be considered the standard of reference and diagnostic accuracy will be evaluated in terms of sensitivity and specificity	15	[98, 99]
12.05.2022 /	Italy	NCT05448157	^68^ Ga-DOTATOC radio-guided surgery with β probe in GEP-NET	Active not recruiting	To evaluate the diagnostic efficacy and the safety of the combined approach with β probe and [^68^ Ga]Ga-DOTA-TOC PET/CT in the correct identification of primary tumor and lymph node metastases, in patients with GEP-NETs candidates to surgery	20	
17.06.2022 /	Belgium	NCT04999917	High-resolution PET-CT Imaging for intraoperative margin assessment in early-stage breast cancer: a prospective multicentric interventional clinical study	Recruiting	Perioperative addressing positive margins of the invasive component in breast cancer	160 (estimated)	[100]
15.02.2023 /	The Netherlands	NCT05446324	Sentinel lymph node mapping with gallium-68-tilmanocept PET/CT in high/high-intermediate risk endometrial cancer: a pilot study	Recruiting	Feasibility of [^68^ Ga]Ga-tilmanocept PET/CT for SLN mapping assessed by the SLN detection rate with ^68^ Ga-tilmanocept PET/CT. Overall SLN detection rate is defined as the proportion of patients in which at least one SLN is detected. Bilateral SLN detection rate is defined as the proportion of patients with at least one SLN detected in each hemipelvis or para-aortic side	10	[20]

Most (10/13) of the noted clinical trials are investigator initiated, almost all (12/13) monocentric and with the objective of validating the technique, rather than comparing to other state-of-the-art techniques. The average number of patients included in a clinical trial is approximately 27 patients.

## Radiation protection and radiation exposure

Where the surgical guidance achieved by using γ-emitting radionuclides has proven to be harmless, the jury is still out regarding the radiation exposure caused by using β-emitting radionuclides. There are many factors that affect the occupational exposure arising from a patient injected with a radiopharmaceutical in the context of surgical radioguidance. In all cases, the ALARA (as low as reasonably achievable) principle should be kept in mind to minimize external radiation exposure to the staff. Unfortunately, the two most effective protective measures, shielding and distance, are hard if not impossible to realize in an operating room setting. For example, for ^18^F, a piece of lead with a thickness of 17 mm would be needed to reduce the exposure to one-tenth [101]. Given that surgery is very much a hands-on intervention, keeping distance is also not an option. In a robot-assisted setting, the operating surgeon is located at a distance from the patient and can potentially even be shielded. However, the anesthesiologist and scrub nurse remain at the patient’s side. Because of these restrictions, there are only two ways to reduce radiation exposure when using β-emitting radionuclides: (1) limit the annual number of exposures per staff member, to keep within the required < 6.0 mSv exposure limits per year (category B workers [102]), or (2) reduce the amount of administered activity used for surgical radioguidance. Examples of radiation exposure of staff during surgical radioguidance with different β-emitting radiopharmaceuticals are presented in Table 4. Reducing procedures’ numbers is highly undesirable given the minimum quota set in some countries [108]; surgeons need to perform a certain number of procedures per year to maintain their license to perform specific types of surgery [109]. Despite the regional differences that are observed in different countries, we propose a volume of 50–100 surgical radioguidance cases a year to be considered surgical routine. Hence, only administered activity reduction remains as a viable option. For β^+^-emitters, to enter the safety range of 150 MBq of ^99m^Tc would mean that about 30 MBq of ^18^F (considering exposure from point source) can be administered, and only 2.3 MBq of ^68^ Ga or ^90^Y can be present in the resected specimen (due to skin exposure from contact) [101, 110]. These values simply offer guidance, as the activity in the patient during surgery is largely determined by tracer pharmacokinetics and the (biological) half-life. Bunschoten et al. presented radiation exposure of patient, surgeon, and non-nuclear personnel [111]. They give an example where they compare radioguidance procedures with ^18^F (370 MBq at 1 h postinjection) resulting in 35 µSv.h^−1^, with a similar procedure with ^99m^Tc (100 MBq at 1 h postinjection) leading to an effective dose rate of 1.9 µSv.h^−1^, considering the average duration of a surgery being 3 h. Here it should be noted that because of their short physical half-life, the effective dose rates of positron emitters will demonstrate a more pronounced decline over time. In the example above, this would result in approximately 62 µSv (^18^F) and 5 µSv (^99m^Tc) for a 3-h cumulative exposure to the reported initial dose rates. Some studies suggest that using a β-emitter purely for radioguided surgery purposes means the injected activity can be reduced down to 1 MBq/kg, thus decreasing the overall exposure [22]. Nevertheless, the use of β-emitters is potentially associated with higher occupational exposure; see also Table 5 and [112, 113].

**Table 4:** Examples of radiation exposure of staff during surgery with β-emitting radiopharmaceuticals

Reference	Povoski et al. [7]	Nalley et al. [103]	Piert et al. [104]	Grootendorst et al. [72]	Collamati et al. [45]	Fragoso Costa et al. [105]	Heuvel et al. [22]	Camillocci et al. [106]	Russomando et al. [107]
Number of patients	10	3	17	22	7	10	6	1	3
Radiopharmaceutical	[^18^F]F-FDG	[^18^F]F-FDG	[^18^F]F-FDG	[^18^F]F-FDG	[^68^ Ga]Ga-PSMA-11	[^68^ Ga]Ga-PSMA-11	[^68^ Ga] Ga-HBEDCC-PSMA	[^90^Y]Y-DOTA-TOC	[^90^Y]Y-DOTA-TOC
Mean administered activity [MBq]	699 ± 181 MBq (preoperative)	280 MBq (preoperative)	52 MBq (preoperative)	295 MBq (preoperative)	70 MBq (intraoperative)	122 MBq (preoperative)	83 MBq (intraoperative)	300 MBq (preoperative)	93–167 MBq (preoperative)
Mean time from administration to surgery	180 min	180 min	184 min	118 min	-	-	73 min	24 h	24 h
Type of surgery	Open	Open	Open	Open	Robot-assisted	Robot-assisted	Robot-assisted	Open	Open
Usage	In vivo	In vivo	In vivo	Ex vivo	Ex vivo	Ex vivo	Ex vivo	Ex vivo	Ex vivo
Maximum effective dose [H_p_(10)] per procedure (µSv)	164 µSv (surgeon)	70 µSv (surgeon)	9 μSv/h (surgeon)	74 μSv/h (surgeon)	16 µSv (scrub nurse)	9 µSv (surgical assistant)	16 µSv (scrub nurse)	< 40 µSv	< 40 µSv
Used dosimeter	OSL (InLight™ dosimeter)	Landauer Luxel	Electronic personal dosimeter (Isotrak DoseGUARD S10)	Electronic personal dosimeter (PDM-112&122)	Electronic personal dosimeter (DMC 2000)	Electronic personal dosimeter	Electronic personal dosimeter (DMC 2000)	Film badge personal dosimeters (AGFA)	Film badge personal dosimeters (AGFA)

**Table 5:** Decay and occupational exposure characteristics of the most widely used radionuclides in surgical radioguidance. Exposure data based in Delacroix 2002 [101]. Exemption levels are based on Council Directive 2013/59/Euratom [102]

Radionuclide	Half-life T_1/2_ [h]	Main decay mode and/or emissions	External exposure—dose-rate constant		Exemption levels	
					Activity concentration [kBq.kg^−1^]	Activity [Bq]
			Point source in 1 m [µSv.m^2^.h^−1^.MBq^−1^] *(deep tissue dose)*	Contact with a 5-mL plastic syringe [mSv.m^2^.h^−1^.MBq^−1^]		
^18^F	1.83	$\beta$^+^	0.163	2.880	10	1×10^6^
^68^ Ga	1.13	$\beta$^+^	0.156	31.400	10	1×10^5^
^124^I	100.32	$\beta$^+^	0.172	10.700	10	1×10^6^
^64^Cu	12.70	$\beta$^+^/$\beta$^−^	0.031	0.579	100	1×10^6^
^90^Y	64.04	$\beta$^−^	0.000	43.500	1000	1×10^5^
^131^I	192.56	$\beta$^−^/$\gamma$	0.066	1.130	100	1×10^6^
^99m^Tc	6.01	$\gamma$	0.024	0.354	100	1×10^7^
^111^In	67.32	$\gamma$	0.089	1.220	100	1×10^6^
^123^I	13.22	$\gamma$	0.046	0.605	100	1×10^7^
^125^I	1437.60	$\gamma$	0.035	0.620	1000	1×10^6^

The dose rate constant is the quantity used for occupational exposure calculations. It represents (in the case of the photons) the ambient dose equivalent H*(10) field flux around a point source and it is specific for each radionuclide; see Table 5 [114]. Personal dosimeters placed on the chest measure the deep dose equivalent H_p_(10) which is usually taken as an operational measure of effective dose for comparison with occupational dose limits. It is also useful, especially for β-emitters, to possess information about the specific dose rate constants regarding the personal surface dose equivalent H_p_(0.07), which is a surrogate for skin exposure. For example, skin exposure rate of a 50-mL source filled with 1 MBq is 0.56 and 0.16 mSv/h for ^18^F and ^99m^Tc, respectively [101].

For the management of surgical waste, superficial contamination must be excluded and exemption levels must be observed [115] (values of activity for which they are exempt from control); see Table 5. The waste that arises during the surgery can in general be liquidated at 10 half-lives after use of radionuclide without radiation measurement. The same exemption levels should be also applied for determining when pathologists can start processing excised tissue specimens, which generally occurs the day after surgery. Semi-conductor germanium detectors can be used for high-precision measurement of specimens to determine if exemption levels are exceeded at the moment of tissue excision [105]. In both the waste management and the pathological processing of tissue, short-lived radionuclides such as ^18^F and ^68^ Ga bring advantages [106]. At the same time, use of longer-lived radionuclides such as ^90^Y and ^89^Zr [18, 107] challenges routine clinical logistics. As much remains unknown, it would be desirable that studies using β-emitting radionuclides be carefully monitored by the medical physicist/radiation safety officer and reported.

## Cost

Perhaps the least popular, but nevertheless a highly relevant, topic is cost. Again, this is an aspect that differs between the use of γ- and β-emitters for radioguidance. Starting with γ-emitters, these tracers are generally relatively low in cost (e.g., 100 € a vial ^99m^Tc-nanocolloid) and intraoperative detection technologies are relatively cheap (e.g., a sterilizable DROP-IN probe costs around 15 k€, including the readout unit). Here, it must be mentioned that the price of receptor-targeted tracers such as [^99m^Tc]Tc-PSMA I&S can be an order of magnitude higher than that of ^99m^Tc-nanocolloid. The cost of a preoperative SPECT/CT roadmap is about 300 €. Moving to β-emitters, 2-[^18^F]FDG is very cost-efficient radiopharmaceutical at around 1 €/MBq, but the cost of a preoperative PET/CT roadmap is expected to be higher than a SPECT/CT (estimated cost 1 k€). The price for other PET tracers, however, tends to vary between 900 € and 3 k€ depending on the radionuclide cost, the common use of the agent, etc. The reimbursement policies for PET and SPECT procedures differ significantly across Europe, depending on the specific healthcare systems in place [116]. For example, in Germany, for an ever-growing number of indications [117], a low-dose PET/CT scan could be reimbursed with rates between 649 € [118] and 775 € [119] plus costs for the radiotracer, whereas a SPECT examination reimburses 110 € [120] plus tracer costs. Being more experimental in nature, also the detector technologies are not yet widely available and acquiring them can end up being more expensive. Prices add to the overall cost of the procedure. Where β-probes could be relatively low in cost [45], clinical Cerenkov specimen imagers’ (product is taken of the market) costs in the range of 100 k€ and hybrid ex vivo PET/CT modalities for specimen imaging go beyond 250 k€. Meaning that for now, radioguidance via β-emitters substantially raises the healthcare costs. For these rises in cost to be valid, the value added by β-surgical radioguidance needs to be related to the (outcome) benefit that patients receive from this form of image-guided surgery. This calls for further cost–benefit analysis, considering improved surgical outcome, potentially lower numbers of futile surgery and early relapses, a decreased requirement for additional systemic treatment, and patient-reported outcomes.

## Ethical aspects

New medical technologies should benefit the patient and the treating physician before finding their way to clinical use. For β-surgical radioguidance, benefit needs to be provided in relation to safe and cost friendly $\gamma$-photon alternatives which generally set the standard in routine care. This raises ethical questions about promoting β-surgical radioguidance technologies without proficiency benchmarking. On one side, one may argue that innovations are the way forward in healthcare; there should be ample opportunity for scientists to gain insight into new technology’s possibilities. On the other side, performance evidence needs to be collected. The International Basic Safety Standards by the IAEA stipulate that “the minimum patient exposure consistent with acceptable image quality can be achieved by appropriate selection of the best available radiopharmaceutical and its activity” [44].The open question then is: Does β-surgical radioguidance strike the right balance? There appear to be pros and cons, but hard clinical evidence is lacking, raising the need for prospective randomized trials that, e.g., compare β-particle- to γ-photon-based surgical guidance technologies. Such trials need approval by local ethic committees and/or competent authorities, require statistically adequate number of procedures, be covered by legislation, provide insurance for patients, and have clear primary and secondary outcomes [121]. If the result of the trials is positive, the benefit still must be outweighed against radiation exposure and costs. Thus, prior to recommendation of β-surgical radioguidance by scientific bodies like EANM, it will have to be evaluated whether the potential benefits exceed the risks of radiation exposure and cost issues.

## Challenges and success drivers

The potential to utilize a single tracer for diagnosis, create a precise surgical roadmap, guide the surgical procedure, and monitor follow-up progress represents a compelling reason to actively pursue β ^+^ /β^−^-radioguidance. But as our summary indicates, this theoretical potential is counterbalanced by substantial practical limitations (e.g., complicated logistics, technical limitations, dose to surgical staff, and cost). Also, important to note here is that to date, evidence has not yet been provided that indicates patient benefit. That said, substantial chemical and technical advances could in the future help address clinical requirements while effectively tackling concerns regarding occupational radiation exposure. In order to hold ground in a more competitive clinical field, it is imperative that these efforts mature and amass collect robust (clinical) evidence. Overall, a critical view of β-surgical radioguidance is required while these cutting-edge technologies mature to a part of clinical routine.

## Liability statement

This paper summarizes the views of the EANM Translational Molecular Imaging & Therapy, Physics, Oncology &Theranostics, Radiation Protection and Ethics Committees. It reflects recommendations for which the EANM cannot be held responsible. The recommendations should be taken into context of good practice of nuclear medicine and do not substitute for national and international legal or regulatory provisions.

## Data Availability

Data will be made available upon request.
